# Abdominal Fat and Metabolic Health Markers but Not PNPLA3 Genotype Predicts Liver Fat Accumulation in Response to Excess Intake of Energy and Saturated Fat in Healthy Individuals

**DOI:** 10.3389/fnut.2020.606004

**Published:** 2020-12-03

**Authors:** Fredrik Rosqvist, Marju Orho-Melander, Joel Kullberg, David Iggman, Hans-Erik Johansson, Jonathan Cedernaes, Håkan Ahlström, Ulf Risérus

**Affiliations:** ^1^Department of Public Health and Caring Sciences, Clinical Nutrition and Metabolism, Uppsala University, Uppsala, Sweden; ^2^Department of Clinical Sciences in Malmö, Lund University, Lund, Sweden; ^3^Department of Surgical Sciences, Radiology, Uppsala University, Uppsala, Sweden; ^4^Antaros Medical AB, BioVenture Hub, Mölndal, Sweden; ^5^Center for Clinical Research Dalarna, Falun, Sweden; ^6^Department of Medical Sciences, Uppsala University, Uppsala, Sweden; ^7^Department of Medical Cell Biology, Uppsala University, Uppsala, Sweden

**Keywords:** liver fat, saturated fat, overfeeding, NAFLD, fatty acids

## Abstract

**Background:** Saturated fat (SFA) has consistently been shown to increase liver fat, but the response appears variable at the individual level. Phenotypic and genotypic characteristics have been demonstrated to modify the hypercholesterolemic effect of SFA but it is unclear which characteristics that predict liver fat accumulation in response to a hypercaloric diet high in SFA.

**Objective:** To identify predictors of liver fat accumulation in response to an increased intake of SFA.

**Design:** We pooled our two previously conducted double-blind randomized trials (LIPOGAIN and LIPOGAIN-2, clinicaltrials.gov NCT01427140 and NCT02211612) and used data from the n = 49 metabolically healthy men (*n* = 32) and women (*n* = 17) randomized to a hypercaloric diet through addition of SFA-rich muffins for 7–8 weeks. Associations between clinical and metabolic variables at baseline and changes in liver fat during the intervention were analyzed using Spearman rank correlation. Linear regression was used to generate a prediction model.

**Results:** Liver fat increased by 33% (IQR 5.4–82.7%; *P* < 0.0001) in response to excess energy intake and this was not associated (*r* = 0.17, *P* = 0.23) with the increase in body weight (1.9 kg; IQR 1.1–2.9 kg). Liver fat accumulation was similar (*P* = 0.28) in carriers (33%, IQR 14–79%) and non-carriers (33%, IQR −11 to +87%) of the PNPLA3-I148M variant. Baseline visceral and liver fat content, as well as levels of the liver enzyme γ-glutamyl transferase (GT), were the strongest positive predictors of liver fat accumulation—in contrast, adiponectin and the fatty acid 17:0 in adipose tissue were the only negative predictors in univariate analyses. A regression model based on eight clinical and metabolic variables could explain 81% of the variation in liver fat accumulation.

**Conclusion:** Our results suggest there exists a highly inter-individual variation in the accumulation of liver fat in metabolically healthy men and women, in response to an increased energy intake from SFA and carbohydrates that occurs over circa 2 months. This marked variability in liver fat accumulation could largely be predicted by a set of clinical (e.g., GT and BMI) and metabolic (e.g., fatty acids, HOMA-IR, and adiponectin) variables assessed at baseline.

## Introduction

The etiology behind excessive accumulation of fat in the liver (non-alcoholic fatty liver disease, NAFLD) is multifactorial and can have metabolic and/or genetic origin. Metabolic NAFLD is associated with an increased risk of type 2 diabetes (1) and cardiovascular disease (2) whereas the primary genetic risk factor identified thus far, the PNPLA3 variant I148M, predisposes to NAFLD without its related metabolic disorders (3). Although obesity, i.e., the consequence of long-term positive energy balance, is an important driver of NAFLD, dietary composition (e.g., types and proportions of fats and carbohydrates) has recently been demonstrated to determine liver fat accumulation independently of changes in body weight (4, 5). Dietary fat composition appears to be of special importance: randomized trials have demonstrated that saturated fat (SFA) promotes and unsaturated fat counteracts liver fat accumulation (6–12). The mechanisms behind the deleterious effects of dietary SFA on liver fat accumulation are elusive and likely multifactorial; possible mechanisms include adipose tissue inflammation leading to increased lipolysis (11), ceramides (8), mitochondrial dysfunction (13), altered gut microbiota (14), and a decreased propensity to enter oxidation pathways compared with unsaturated fat (15). Notably, results from intervention trials demonstrate that the inter-individual change in liver fat accumulation in response to dietary SFA is quite considerable (6–8, 10), implying that phenotypic and/or genotypic characteristics exert strong modulatory effects. Such individual responses to SFA have previously been demonstrated for blood lipids, where the effect of dietary SFA on LDL cholesterol and apolipoprotein B levels are about half the magnitude in subjects with obesity compared with normal-weight subjects (16, 17). Similarly, insulin sensitivity may be a determinant of the effects of dietary modifications (18, 19). However, whether body fatness, insulin resistance or other phenotypic characteristics determines the stimulatory effect of dietary SFA on liver fat accumulation is unknown. Thus, the aim of the present study was to identify metabolic and genetic predictors of liver fat accumulation in response to an increased dietary hypercaloric intake of SFA in humans.

## Subjects and Methods

### Subjects

We used data from our two previously conducted randomized trials LIPOGAIN (7) and LIPOGAIN-2 (8) comparing the effects of SFA and polyunsaturated fat during excess energy intake. Subjects in both trials were healthy men and women recruited from the general population using local advertisements. The dietary interventional protocol and methods were identical and performed at the same clinic with all analyses and assessment performed at the same laboratory and equipment. For both trials, the main finding was that a hypercaloric intake of SFA-rich muffins resulted in increased liver fat content whereas a hypercaloric intake of muffins rich in polyunsaturated fat did not change liver fat content despite similar body weight gain (7, 8). Thus, the composition of dietary fat determined whether liver fat increased or not, during concomitant and similar intake of carbohydrates, sugars and total excess energy. For the present study, we pooled the SFA arms from both studies resulting in *n* = 49 subjects (*n* = 20 from LIPOGAIN and *n* = 29 from LIPOGAIN-2) consuming diets enriched with muffins particularly high in SFA in addition to carbohydrates, for 7–8 weeks. LIPOGAIN was carried out between August through December 2011 and LIPOGAIN-2 was carried out between August 2014 through June 2015, both at Uppsala University Hospital, Uppsala, Sweden.

### Dietary Intervention

Subjects received muffins baked with fat from palm oil (high in SFA) to consume in addition to their habitual diet, aiming for an increase in body weight by 3%. The nutrient composition of the muffins was 51% of energy from fat (of which 23E% from SFA, 19E% from MUFA and 5.5E% from PUFA), 5% from protein and 44% from carbohydrates (sugar:starch ratio 55:45). One muffin contained ca 250 kcal and the participants consumed on average ca 3 muffins/day. The muffins were identical in both studies but the duration of the intervention was 7 weeks in LIPOGAIN and 8 weeks in LIPOGAIN-2; as previously described in detail (7, 8). Dietary intake was assessed by 4-day weighed food records at baseline and at the end of the intervention and processed using DietistXP v3.1 (Kost och Näringsdata, Bromma, Sweden).

### Liver Fat, Clinical, and Metabolic Variables

Details of the methods have previously been reported (7, 8). Briefly, liver fat, visceral adipose tissue (VAT), and body composition were assessed using MRI, clinical chemistry was analyzed using routine methods at the hospital and fatty acid composition was analyzed using gas chromatography. Waist circumference was measured midway between the lowest ribs and the iliac crest, in standing position after a normal exhalation.

### Genotyping

The *PNPLA3* rs738409 (Ile148Met, C/G) was genotyped using the Taqman PCR method (Applied Biosystems, Foster City, CA USA), according to the manufacturer's instructions. ABI Prism Sequence Detection Systems ABI 7900HT (Applied Systems) was used for post-PCR allelic discrimination by allele-specific fluorescence. Genotypes were successfully determined for all individuals, and concordance rate of repeated genotyping of all individuals was 100%.

### Statistics

Subgroups based on the change in liver fat content (above or below median) were compared using Wilcoxon test. Associations between baseline characteristics and change in liver fat content were analyzed using Spearman rank correlation. *P* < 0.05 (univariate) and *P* < 0.01 (regression model) was considered statistically significant. All variables significantly associated with liver fat accumulation in univariate analyses were initially included in the regression model. To avoid multicollinearity, the variable list was sequentially culled based on the highest variance inflation factor (VIF) until all included variables had a VIF <5. The variable list was then further culled based on the highest *P*-value until all included variables had a *P* < 0.01. Univariate analyses were performed in the whole cohort (*n* = 49) as well as in men (*n* = 32) and women (*n* = 17) separately, whereas the regression model was built in the whole cohort only due to low sample size. No adjustment was made for multiple comparisons. Data are given as median (IQR) and were analyzed using JMP 14.1.0 (SAS, Cary, NC, USA).

## Results

Baseline characteristics of the subjects are shown in Table 1.

**Table 1:** Baseline characteristics.

	**All (*n* = 49)**	**Low liver fat change (<median, *n* = 25)**	**High liver fat change (≥median, *n* = 24)**	*P*-value^*^
Sex (M/F)	32/17	15/10	17/7	0.55
Age, y	34 (27–45)	29 (26–42)	42 (28–46)	0.05
BMI	25.6 (20.0–28.2)	23.8 (20.2–26.6)	27.3 (19.9–29.1)	0.11
Waist, cm	86.3 (78.1–99.0)	81.5 (75.8–93.0)	97.0 (80.0–103.0)	0.02
Liver fat, %	1.1 (0.9–2.3)	1.0 (0.85–1.4)	1.5 (0.9–4.8)	0.03
Systolic BP, mm Hg	120 (112–127)	117 (111–125)	121 (114–137)	0.09
Diastolic BP, mm Hg	76 (70–82)	73 (70–78)	78 (70–86)	0.04
CRP, mg/L	0.9 (0.4–2.3)	0.7 (0.4–1.9)	1.2 (0.3–2.5)	0.67
LDL cholesterol, mmol/L	2.5 (2.2–3.1)	2.4 (2.1–2.9)	2.6 (2.2–3.2)	0.33
HDL cholesterol, mmol/L	1.3 (1.1–1.5)	1.3 (1.2–1.5)	1.2 (1.0–1.5)	0.14
Triglycerides, mmol/L	0.7 (0.6–1.1)	0.7 (0.6–0.9)	0.9 (0.6–1.2)	0.14
Glucose, mmol/L	5.2 (4.7–5.8)	4.9 (4.5–5.6)	5.5 (5.0–5.8)	0.04
Insulin, mU/L	6.8 (5.1–8.7)	6.3 (4.1–8.1)	7.4 (5.8–11.1)	0.13
HOMA-IR	1.6 (1.1–2.1)	1.3 (0.9–2.1)	1.7 (1.4–2.7)	0.08
Adiponectin, μg/mL	0.33 (0.23–6.22)	5.38 (0.26–7.73)	0.25 (0.18–3.98)	0.007
PNPLA3 II/MI/MM	25/19/5	16/7/2	9/12/3	0.17
Energy, kcal	2,490 (1,845–2,951)	2,494 (1,906–2,758)	2,410 (1,705–3,161)	0.99
Carbohydrate, E%	42.0 (38.3–48.0)	42.0 (38.0–50.0)	41.5 (38.3–47.5)	0.80
Protein, E%	15.9 (14.0–17.2)	16.0 (14.0–17)	15.8 (14.0–17.7)	0.77
Fat, E%	37.1 (31.7–41.1)	36.9 (32.0–40.8)	37.6 (31.1–42.4)	0.67
SFA, E%	13.9 (11.8–16.6)	13.9 (11.5–16.3)	15.1 (12.2–17.8)	0.42
MUFA, E%	13.3 (10.7–16.2)	12.9 (10.1–16.0)	13.4 (10.9–16.4)	0.56
PUFA, E%	4.9 (3.7–6.1)	4.9 (3.5–6.2)	4.8 (3.8–6.1)	1.0

* *Comparing the subgroups with smaller and larger changes in liver fat using Wilcoxon test. Data are median (IQR). BMI, body mass index; BP, blood pressure; CRP, C-reactive protein; E%, percent energy; LDL, low density lipoprotein; HDL, high density lipoprotein; HOMA-IR, homeostatic model assessment of insulin resistance; SFA, saturated fatty acids; MUFA, monounsaturated fatty acids; PUFA, polyunsaturated fatty acids; PNPLA3, patatin-like phospholipase domain-containing protein 3*.

### Dietary Intake

Dietary intake of SFA increased by a median of 3.5% energy (%E, percent of total energy intake) (IQR 1.1–6.0%E, *P* < 0.0001) during the intervention, from 13.9%E (IQR 11.8–16.6%E) at baseline to 17.2%E (IQR 15.3–19.5%E) at the end of the intervention. Total energy intake increased by a median of 686 kcal (IQR 89–833 kcal, *P* < 0.0001), from 2,490 kcal (IQR 1,845–2,951 kcal) at baseline to 2,986 kcal (IQR 2,564–3,289 kcal) at the end of the intervention. Intake of total fat increased by a median of 3%E (IQR 1.2–7.7%E, *P* < 0.0001), from 37.1%E (IQR 31.7–41.1%E) at baseline to 40.0%E (IQR 37.1–44.1%E) at the end of the intervention whereas intake of protein decreased by a median of 3.0%E (IQR 4.8–0.7%E, *P* < 0.0001), from 15.9%E (IQR 14.0–17.2%E) at baseline to 13.0%E (IQR 11.2–15.1%E) at the end of the intervention. In contrast, dietary intake of total carbohydrates did not change during the intervention [42.0%E (IQR 38.3–48.0%E) at baseline, 41.5%E (IQR 39.0–47.0%E) at the end of the intervention, *P* = 0.34]. Data on total sugar intake was unfortunately not available.

### Liver Fat Accumulation

**Figure 1:**
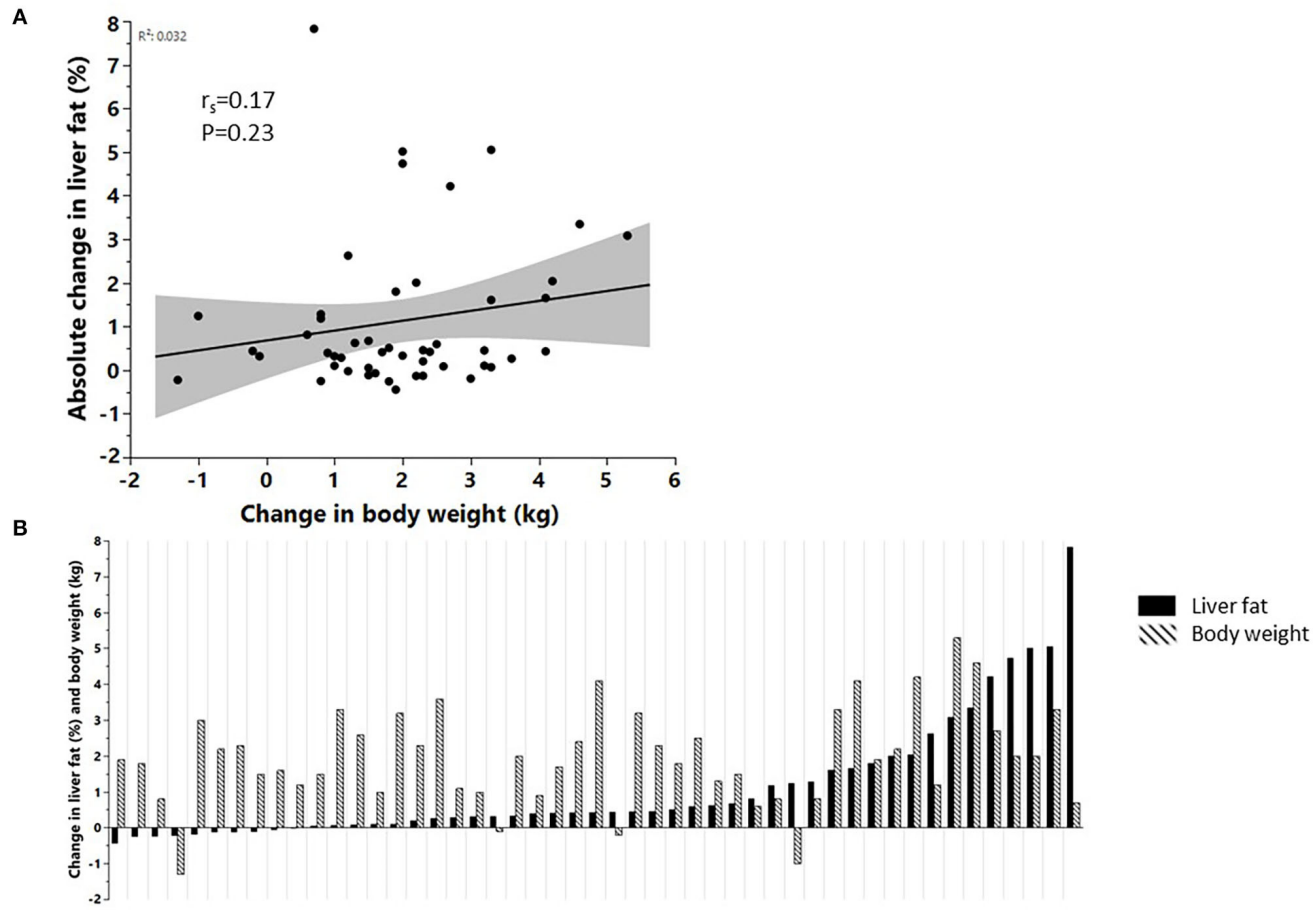
**(A)** Correlation between the change in body weight and change in liver fat accumulation (*n* = 49), **(B)** changes in liver fat content and body weight at the individual level, sorted according to the relative change in liver fat content (*n* = 49).

In the whole cohort (*n* = 49), liver fat content increased by a relative 33% (IQR 5.4–82.7%; *P* < 0.0001) in response to SFA, but this increase was not associated with the increase in body weight (1.9 kg; IQR 1.1–2.9 kg) (Figures 1A,B). Similarly, the increase in liver fat was not associated with an increase in total adipose tissue mass (*r* = 0.15, *P* = 0.30) or abdominal subcutaneous adipose tissue mass (*r* = 0.22, *P* = 0.12). However, there was an association between increased liver fat and increased VAT mass (*r* = 0.4, *P* = 0.005). At baseline, *n* = 5 subjects had NAFLD (defined as liver fat content ≥5.5%) whereas *n* = 10 subjects had NAFLD at the end of the intervention. Carriers of the PNPLA3-I148M variant (MI + MM, *n* = 24) showed a similar (*P* = 0.28) relative increase in liver fat [33.3% (13.9–78.6%) compared with non-carriers (33.3% (−10.7 to 87.0%), *n* = 25].

When subjects were split into two groups by median increase in liver fat, sex distribution, and BMI were similar between groups but glucose, diastolic blood pressure, and liver fat content at baseline were higher in subjects with a larger compared with smaller increase in liver fat content (Table 1). Age tended to be higher (42 vs. 29, *P* = 0.05) in subjects with a larger compared to smaller increase in liver fat (Table 1). In response to consumption of SFA, these two groups increased similar in body weight but differed in the accumulation of liver fat and VAT mass, as well as levels of insulin and liver enzymes (Table 2).

**Table 2:** Changes in clinical and metabolic variables in the subgroups with low and high liver fat change, in response to increased intake of SFA.

	**Low liver fat change (<median)**	**High liver fat change (≥median)**	***P*-value**
Body weight, kg	1.7 (1.0 to 2.4)	2.1 (1.2 to 3.3)	0.16
Waist, cm	2.5 (0.5 to 4)	2 (1 to 5)	0.87
Liver fat, %	0.1 (−0.1 to 0.3)	1.6 (0.6 to 3.3)	<0.0001
VAT, L	0.19 (0.08 to 0.34)	0.35 (0.17 to 0.60)	0.01
TAT, L	1.7 (1.0 to 2.2)	2.1 (1.5 to 2.5)	0.23
Pancreas fat, %	−0.3 (−1.1 to 0.7)	0.7 (−0.5 to 1.9)	0.11
Lean tissue (MRI), L	0.3 (−0.1 to 0.8)	0.5 (−0.3 to 1.4)	0.34
Triglycerides, mmol/L	−0.1 (−0.2 to 0.0)	0.0 (−0.1 to 0.4)	0.05
Insulin, mU/L	−0.25 (−1.2 to 1.4)	1.4 (−0.2 to 3.5)	0.01
Glucose, mmol/L	0.1 (−0.2 to 0.2)	0.1 (−0.2 to 0.2)	0.67
HOMA-IR	−0.1 (−0.3 to 0.3)	0.3 (0.0–0.9)	0.01
ALT, μkat/L	0.02 (−0.05 to 0.05)	0.11 (−0.04 to 0.23)	0.03
γGT, μkat/L	0.0 (−0.02 to 0.01)	0.04 (−0.04 to 0.08)	0.06
Adiponectin, μg/mL	0.07 (−0.02 to 0.39)	0.02 (−0.01 to 0.08)	0.20

*Data are median (IQR) and compared using Wilcoxon test. ALT, alanine aminotransferase; GT, glutamyltransferase; HOMA-IR, homeostatic model assessment of insulin resistance; MRI, magnetic resonance imaging; VAT, visceral adipose tissue; TAT, total adipose tissue*.

### Predictors of Liver Fat Accumulation

**Figure 2:**
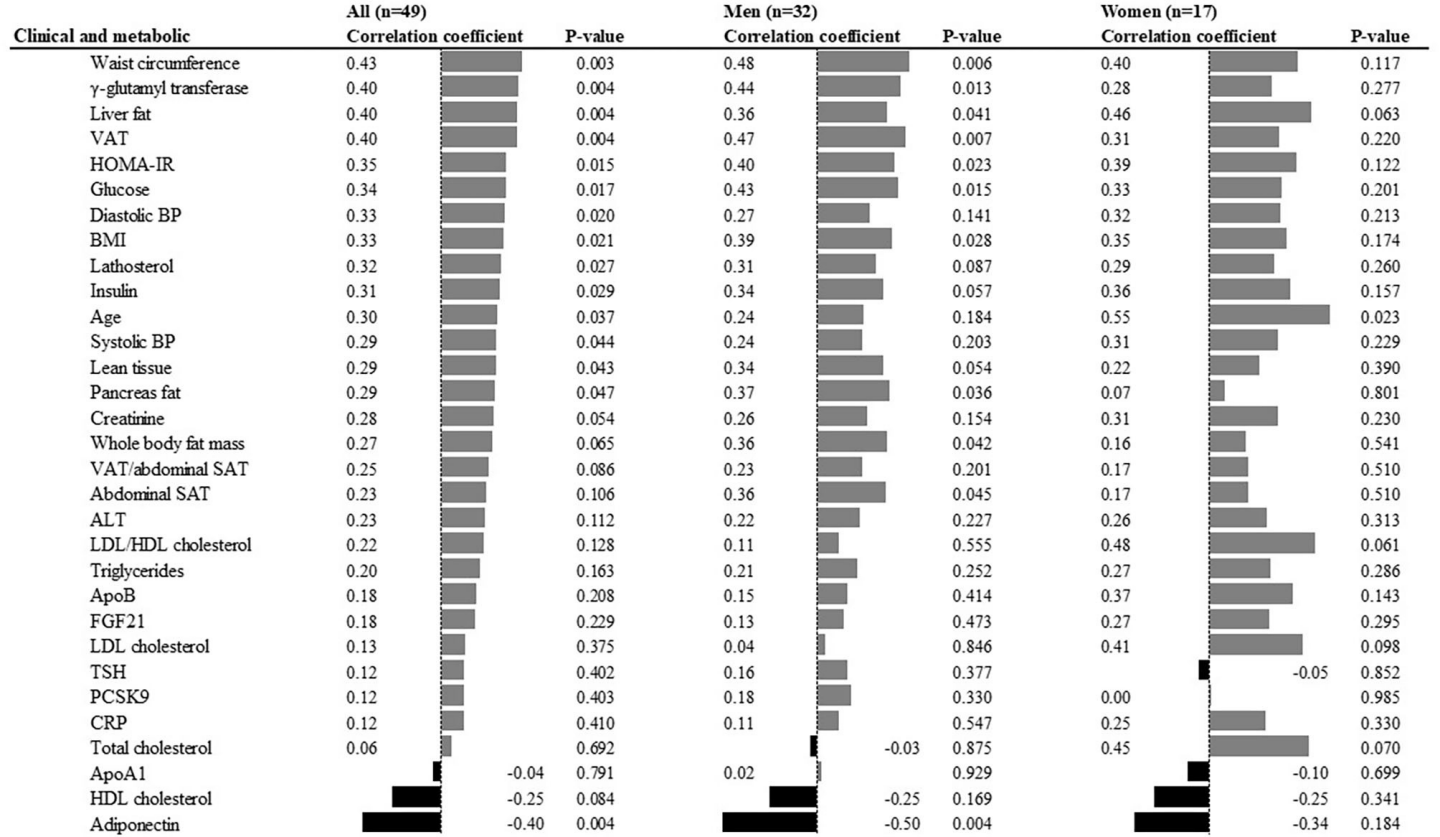
Clinical and metabolic predictors of liver fat accumulation in univariate analyses. ALT, alanine aminotransferase; BMI, body mass index; HOMA-IR, homeostatic model assessment of insulin resistance; LDL, low density lipoprotein; HDL, high density lipoprotein; Apo, apolipoprotein; VAT, visceral adipose tissue; SAT, subcutaneous adipose tissue; BP, blood pressure; FGF21, fibroblast growth factor; TSH, thyroid stimulating hormone; PCSK9, proprotein convertase subtilisin/kexin type 9; CRP, C-reactive protein.

**Figure 3:**
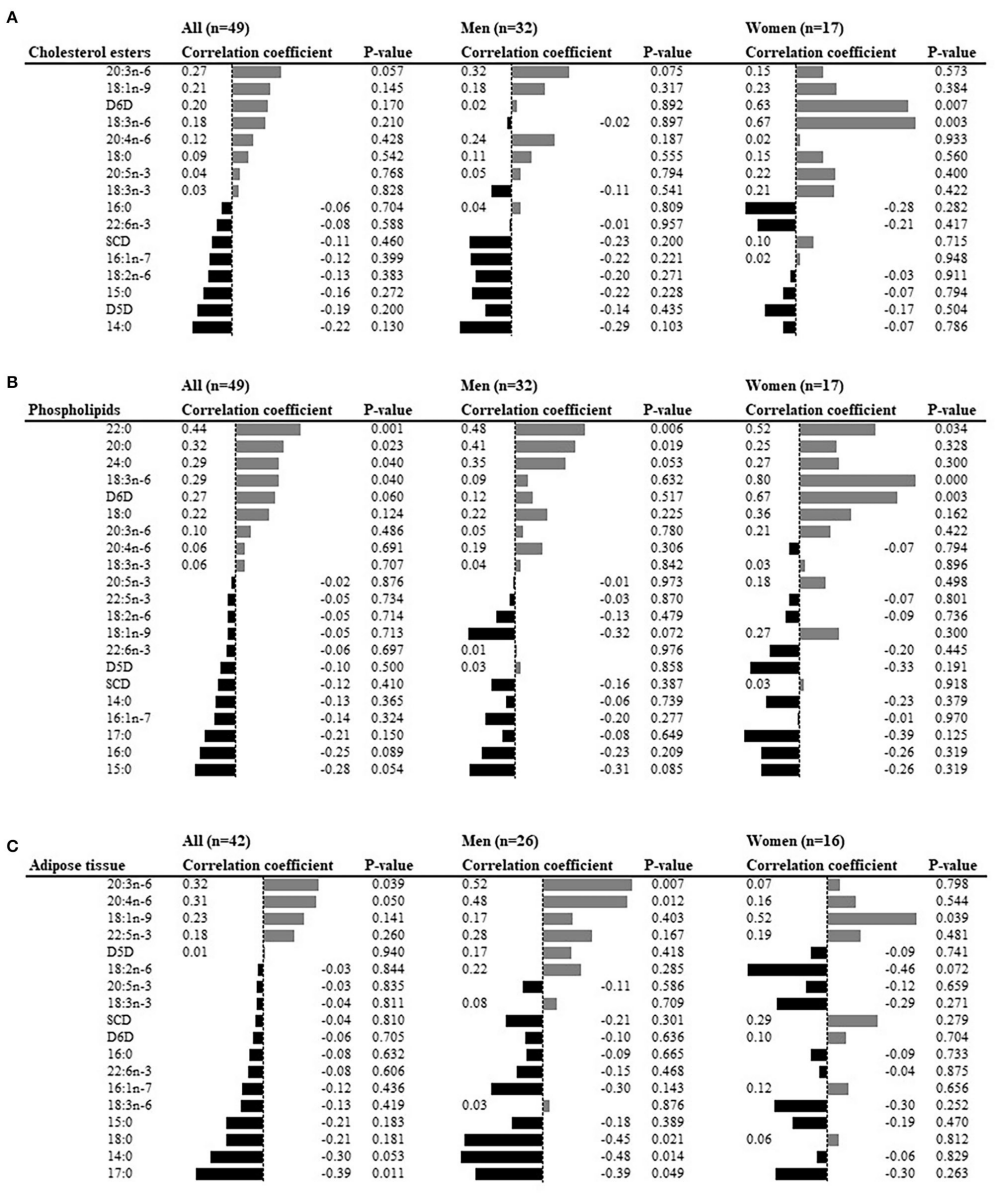
Fatty acid abundances at baseline as predictors of liver fat accumulation in univariate analyses. **(A)** Plasma cholesterol esters, **(B)** plasma phospholipids, **(C)** subcutaneous adipose tissue. The bars represent the Spearman rank correlation (written beside the bars). SCD; stearoyl-CoA desaturase; D6D, delta-6 desaturase; D5D, delta-5 desaturase.

To identify predictors of liver fat accumulation, the associations between clinical and metabolic characteristics at baseline, and the change in liver fat content, were analyzed. Waist circumference, γ-glutamyl transferase, liver fat, VAT, total body fat, and HOMA-IR were the six top positive predictors, whereas adiponectin was the only negative predictor (Figure 2). Associations were overall directionally similar in men and women (Figure 2). Of the fatty acid levels at baseline, higher levels of very long-chain SFA (20:0, 22:0, 24:0) and 18:3n-6 in plasma phospholipids, 20:3n-6 and 20:4n-6 in adipose tissue, were positive predictors. In contrast, the level of 17:0 in adipose tissue was the only negative predictor (Figures 3A–C) in the whole cohort. Sexual dimorphism was observed for some fatty acids: the levels of 18:3n-6 in both phospholipids and cholesterol esters demonstrated strong associations with liver fat accumulation (*r* = 0.80 and *r* = 0.67, respectively) in women but not in men (Figures 3A,B). Furthermore, in adipose tissue, the levels of 20:3n-6 and 20:4n-6 were positively associated with liver fat accumulation in men but not women, and 18:1n-9 was positively associated in women but not men, whereas an inverse correlation was seen between liver fat accumulation and the levels of 14:0 and 18:0 in men but not women (Figure 3C).

**Figure 4:**
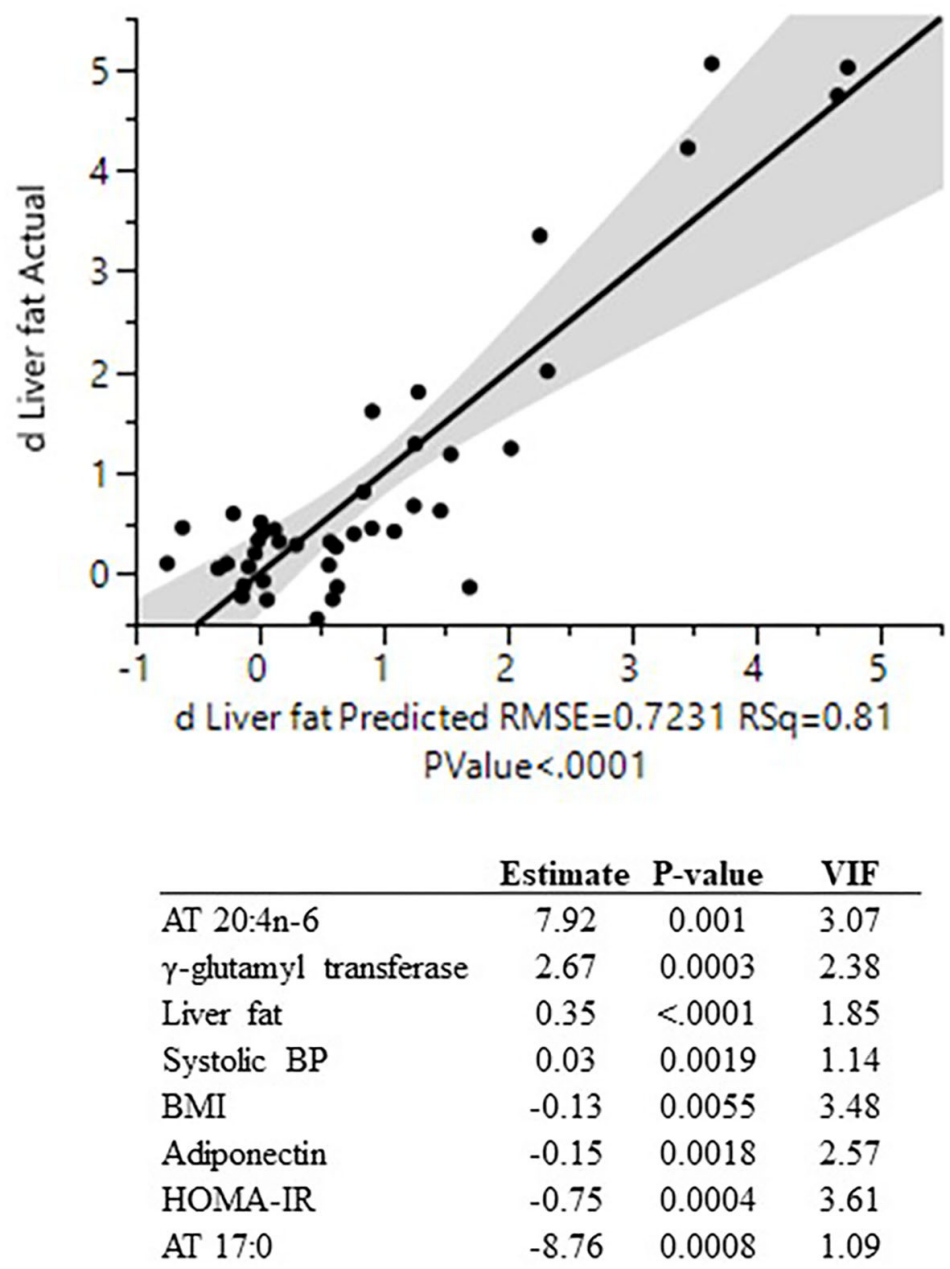
Actual by predicted plot (linear regression) for change in liver fat content and the eight individual predictors used in the regression model. All variables significantly associated with liver fat accumulation in univariate analyses were initially included in the regression model. To avoid multicollinearity, the variable list was sequentially culled based on the highest variance inflation factor (VIF) until all included variables had a VIF <5. The variable list was then further culled based on the highest *P*-value until all included variables had a *P* < 0.01. AT, adipose tissue; BP, blood pressure; VIF, variance inflation factor; HOMA-IR, homeostatic model assessment of insulin resistance; BMI, body mass index.

The linear regression analysis (based on eight variables, using the whole cohort) could explain 81% of the variation in liver fat change (Figure 4) with the following prediction model: Δ liver fat = 0.927 + (7.92^*^20:4n-6_adipose_tissue_) + (2.67^*^γGT) + (0.35^*^liver fat_baseline_) + (0.03^*^systolic BP) – (0.13^*^BMI) – (0.15^*^adiponectin) – (0.75^*^HOMA-IR) – (8.76^*^17:0_adipose_tissue_).

## Discussion

We demonstrate a large inter-individual variation in the accumulation of liver fat in metabolically healthy men and women in response to a hypercaloric diet high in SFA, based on data from two similar studies designed to achieve excess energy intake over a circa 2-month-long period. Notably, this marked variability in liver fat accumulation was not explained by changes in body weight or by presence of the PNPLA3-I148M variant. Visceral and liver fat content at baseline, as well as levels of the liver enzyme γ-glutamyl transferase, were the strongest positive predictors of liver fat accumulation. In contrast, levels of adiponectin in plasma and the fatty acid 17:0 in adipose tissue were the only negative predictors. Using linear regression, a combination of eight clinical and metabolic predictive variables could explain as much as 81% of the variation in liver fat accumulation in response to an increased intake of SFA.

Our finding of high variability in liver fat accumulation induced by overfeeding a diet high in SFA is novel, and points toward a notable modulation by individual phenotypic characteristics. It has previously been reported that the effect of dietary SFA on atherogenic lipids in subjects with obesity was only about ~50% of the effect observed in subjects with normal body weight (16, 17), but no data on liver fat content *per se* has been reported. In response to intentional weight gain achieved by overconsumption of fast food (typically high in SFA), the metabolic response was dependent on the metabolic state at baseline, i.e., only metabolically “unhealthy” subjects showed deteriorations in hepatic-, skeletal muscle-, and adipose tissue insulin sensitivity whereas metabolically “healthy” subjects were protected despite similar increases in body weight and fat mass (20). Similarly, the absolute increase in liver fat accumulation was considerably higher in metabolically “unhealthy” compared with “healthy” subjects (20), but no individual predictors were investigated. In the present study in metabolically healthy subjects fed a hypercaloric diet, a relatively large proportion of subjects showed no or only a very modest increase in liver fat content, despite a significant increase in body weight, whereas others showed a substantial gain in liver fat. Taken together, this implies that although consumption of SFA that exceeds energy demands results in greater liver fat accumulation at the group level, as consistently shown in several trials (6–8, 10, 11), this might partly be driven by a subset of hyper-responders, whereas others show a more neutral response.

An increased understanding of which individual characteristics that determine the harmfulness of dietary SFA is needed for the development of effective personalized nutrition approaches. It has been proposed that increased adipose tissue capacity for lipogenesis (and thus adipose tissue expandability) is protective against adverse metabolic effects of weight gain such as ectopic fat accumulation (20). However, in the present study, there were no associations between increases in total- or abdominal subcutaneous adipose tissue mass, and the increase in liver fat content. Instead, we found that measures of central adiposity (waist circumference and VAT) and liver fat content at baseline were positively associated with further increases in liver fat content during SFA-induced weight gain. Interestingly, the simple measure waist circumference was one of the strongest predictors of liver fat, equal in its predictive magnitude to that observed for total fat mass and even VAT measured by MRI, and thus a finding of clinical interest. The predictive role of waist circumference on liver fat has been reported in several studies (21). It has been observed previously that subjects with NAFLD and prediabetes show larger increases in liver fat content in response to weight gain induced by a SFA-rich diet compared with metabolically healthy subjects with low liver fat content (20). Here we thus extend this finding by showing that liver fat content and central adiposity are positive predictors of liver fat accumulation also in metabolically healthy subjects without NAFLD.

Furthermore, we found that the levels of several fatty acids, in different compartments, were associated with liver fat accumulation. In fact, when using a multiple linear regression model, the strongest positive and negative predictors in the final model were indeed the levels of fatty acids. This model could explain a large proportion (81%) of the variation in liver fat accumulation, with the abundance of the fatty acids 20:4n-6 and 17:0 in adipose tissue constituting the strongest positive and negative contributors, respectively.

It is noteworthy that, in univariate analyses, the levels of very long-chain SFAs (20:0, 22:0, and 24:0) in plasma phospholipids were positively associated with liver fat accumulation. The levels of these very long-chain SFAs have previously been inversely associated with type 2 diabetes (22) and mortality (23). What variation in levels of these particular fatty acids actually represent is unclear but may reflect phenotypic or genotypic characteristics that were not assessed in the current study, such as an intrinsic or environmentally regulated capacity for fatty acid elongation. Another noteworthy finding was that levels of 17:0 in adipose tissue, a biomarker of dairy fat intake and predictor of lower risk of type 2 diabetes (24) was the strongest inverse predictors of liver fat accumulation. The strong sexual dimorphism observed for the predictive role of levels of some fatty acids (e.g., 18:3n-6) was intriguing but should be interpreted with caution, given the smaller sample sizes in the sex-specific analyses.

Fatty acid composition is affected not only by diet and metabolic state but also by genetic variation, in e.g., desaturases (25, 26) and PNPLA3 (27). The PNPLA3-I148M variant is the major genetic risk factor for NAFLD and is known to alter fatty acid composition in multiple compartments (27–29). The PNPLA3-I148M variant affects the hepatic metabolism of PUFA but cell studies indicate no differential effects on the metabolism of SFA (27), congruent with the lack of an effect on the magnitude of liver fat accumulation in response to SFA in the current study. Information on genetic variation in other genes related to fatty acid metabolism, e.g., fatty acid desaturases and elongases, was not available and a limitation of the present study. Other limitations include the homogenous study population, e.g., subjects were overall metabolically healthy and the majority did not have NAFLD. Furthermore, the muffins also contained refined carbohydrate/sugar. Therefore, the effect on liver fat accumulation cannot be ascribed to the intake of SFA alone, but may rather be the result of an interaction between excess sugar and SFA intake (30). Thus, the prediction model generated herein may mainly be valid during overconsumption of both SFA and sugars/refined carbohydrates. Indeed these two macronutrients often come together in commonly consumed junk foods, sweets and bakery. However, the overall intake of carbohydrates did not change during the intervention suggesting that SFA is the main driver of liver fat accumulation, as previously suggested (11). Finally, statistical power and chance findings should be considered in these exploratory analyses and results should be interpreted cautiously. Potential sex-specific predictors should be further investigated and the prediction expression presented herein should be tested, and ideally simplified, in further studies. A strength of the current study is the inclusion of both men and women, whom were overall highly adherent to a standardized dietary overfeeding protocol. In addition, the extensive and broad metabolic assessment allowed for detailed phenotypic characterization of the subjects. However, the findings presented herein would need to be replicated in larger and more diverse populations, e.g., in other randomized trials or in prospective cohort studies with repeated assessments.

In conclusion, we demonstrate substantial inter-individual variation in the accumulation of liver fat in metabolically healthy men and women, in response to an excess energy intake from mainly SFA and carbohydrates. This marked variability in liver fat accumulation was not explained by changes in body weight but could largely be predicted by a set of clinical and metabolic variables that were assessed at baseline.

## Data Availability Statement

The datasets presented in this study can be found in online repositories. The names of the repository/repositories and accession number(s) can be found at: https://osf.io/2ma7e/.

## Ethics Statement

The studies involving human participants were reviewed and approved by Regional Ethical Review Board in Uppsala (Dnr 2011/095) Regional Ethical Review Board in Uppsala (Dnr 2014/186). The patients/participants provided their written informed consent to participate in this study.

## Author Contributions

FR conducted the LIPOGAIN trials, performed statistical analyses, and wrote the first draft. MO-M performed genotyping. JK conducted MRI analyses. DI, H-EJ, and JC obtained biopsies. HA conducted MRI analyses. UR was the principal investigator for the LIPOGAIN trials and conceived the initial idea. All authors reviewed and approved the final manuscript.

## Conflict of Interest

The authors declare that the research was conducted in the absence of any commercial or financial relationships that could be construed as a potential conflict of interest.
